# The Cardiac Ventricular 5-HT_4_ Receptor Is Functional in Late Foetal Development and Is Reactivated in Heart Failure

**DOI:** 10.1371/journal.pone.0045489

**Published:** 2012-09-20

**Authors:** Trond Brattelid, Eirik Qvigstad, Lise R. Moltzau, Silje V. S. Bekkevold, Dagny L. Sandnes, Jon Arne K. Birkeland, Tor Skomedal, Jan-Bjørn Osnes, Ivar Sjaastad, Finn Olav Levy

**Affiliations:** 1 Department of Pharmacology, Faculty of Medicine, University of Oslo and Oslo University Hospital, Oslo, Norway; 2 Center for Heart Failure Research, Faculty of Medicine, University of Oslo, Oslo, Norway; 3 National Institute of Nutrition and Seafood Research, Bergen, Norway; 4 Institute for Experimental Medical Research, University of Oslo and Oslo University Hospital, Oslo, Norway; 5 Department of Cardiology, Oslo University Hospital - Ullevaal, Oslo, Norway; 6 K. G. Jebsen Cardiac Research Centre, Oslo, Norway; Centro Cardiologico Monzino, Italy

## Abstract

A positive inotropic responsiveness to serotonin, mediated by 5-HT_4_ and 5-HT_2A_ receptors, appears in the ventricle of rats with post-infarction congestive heart failure (HF) and pressure overload-induced hypertrophy. A hallmark of HF is a transition towards a foetal genotype which correlates with loss of cardiac functions. Thus, we wanted to investigate whether the foetal and neonatal cardiac ventricle displays serotonin responsiveness. Wistar rat hearts were collected day 3 and 1 before expected birth (days -3 and -1), as well as day 1, 3, 5 and 113 (age matched with Sham and HF) after birth. Hearts from post-infarction HF and sham-operated animals (Sham) were also collected. Heart tissue was examined for mRNA expression of 5-HT_4_, 5-HT_2A_ and 5-HT_2B_ serotonin receptors, 5-HT transporter, atrial natriuretic peptide (ANP) and myosin heavy chain (MHC)-α and MHC-β (real-time quantitative RT-PCR) as well as 5-HT-receptor-mediated increase in contractile function *ex* *vivo* (electrical field stimulation of ventricular strips from foetal and neonatal rats and left ventricular papillary muscle from adult rats in organ bath). Both 5-HT_4_ mRNA expression and functional responses were highest at day -3 and decreased gradually to day 5, with a further decrease to adult levels. In HF, receptor mRNA levels and functional responses reappeared, but to lower levels than in the foetal ventricle. The 5-HT_2A_ and 5-HT_2B_ receptor mRNA levels increased to a maximum immediately after birth, but of these, only the 5-HT_2A_ receptor mediated a positive inotropic response. We suggest that the 5-HT_4_ receptor is a representative of a foetal cardiac gene program, functional in late foetal development and reactivated in heart failure.

## Introduction

The mRNA expression of several cardiac G-protein-coupled receptors is altered in heart failure (HF) [1]. A hallmark of the cardiac remodelling in heart failure is activation of a foetal gene program, which reflects an adaptive or possibly maladaptive response to various pathophysiological stimuli such as haemodynamic stress and altered neurohumoral activation [2]. Characteristic changes observed in the failing heart such as a switch from myosin heavy chain (MHC)-α to MHC-β [3], altered fatty acid and glucose energy metabolism [4] and increased production of natriuretic peptides are all features of a foetal phenotype.

Detection of genes encoding proteins involved in foetal cardiac development should enable a better understanding of cardiac remodelling as they are involved in neurohumoral signalling activated during heart failure. Components of the foetal gene program, reactivated in heart failure, represent potential therapeutic targets in the treatment of heart failure [5].

Serotonin (5-HT) is a key molecule in the early embryogenesis [6] and acts as a trophic signal during embryonal heart development [7]. However, although direct cardioexcitation by 5-HT was initially not observed in the normal adult cardiac ventricle in either man [8], [9] or rat [10], we found an upregulated 5-HT_4_ receptor mRNA level accompanied by a 5-HT_4_-mediated positive inotropic response to 5-HT in the cardiac ventricle of failing human hearts [11] as well as both infarcted, failing and hypertrophic rat hearts [12], [13]. Interestingly, coronary artery disease and heart failure is associated with an increase in the plasma level of 5-HT [14], [15], [16], [17]. The 5-HT_4_ receptor might also be a potential therapeutic target, and long-term addition of the potent, selective 5-HT_4_ receptor antagonist piboserod to standard treatment in patients with chronic HF increased left ventricular ejection fraction (LVEF) compared to standard treatment alone [18]. In acute rat heart failure robust cardioexcitatory effects of serotonin rapidly appear and levels of mRNA encoding both 5-HT_4_ and 5-HT_2A_ increase [19]. Thus, in the cardiac ventricle the transition from a non-diseased heart to a failing phenotype is accompanied by substantial alterations in the 5-HT signalling system.

The recognition that genes activated during HF may be a part of a late foetal gene program dominated by hypertrophic growth and that 5-HT plays a trophic role in cardiac embryonal development [7] implies a potential role of 5-HT receptors in the function of both the foetal and failing heart. Thus the induction of serotonin responsiveness during heart failure may represent a reactivation of a foetal gene expression pattern.

We here demonstrate that 5-HT_4_ receptor mRNA expression and 5-HT_4_-mediated inotropic response are augmented, not only in HF, but also in late foetal development, indicating that the 5-HT_4_ receptor gene is a representative of a late stage foetal cardiac gene program. In addition, we found that both 5-HT_2A_ and 5-HT_2B_ receptor mRNAs were increased at the time of birth, but only the former mediated an inotropic response.

## Materials and Methods

The study conforms to the Guide for the Care and Use of Laboratory Animals published by the US National Institutes of Health (NIH Publication No. 85–23, revised 1996) and the Norwegian National Guidelines for Research Ethics in Science and Technology. The experimental protocol was approved by the Norwegian Animal Research Authority (approval ID 2/05), and all procedures described were performed in accordance with their recommendations.

Pregnant rats and mothers with litters were kept in separate cages whereas two adult male rats were kept in each cage and housed in a temperature-regulated room on 12 h:12 h day/night cycle. The animals were given access to food and water *ad libitum*.

Heart tissue was collected from of foetal rats 3 days and 1 day before expected birth (days -3 and -1) and neonatal rats 1, 3 and 5 days after birth (days 1,3 and 5) as well as from 113-day-old rats (age matched with Sham and HF rats 6 weeks after infarction as described below), used as adult controls. An extensive myocardial infarction was induced by coronary artery ligation as described [20] in male Wistar rats (320 g; Møllegård Breeding and Research Center, Skensved, Denmark) anaesthetised with 68% N_2_O, 29% O_2_ and 2–3% isoflurane (Abbot Park, Illinois, USA), and ventilated on a respirator (Zoovent, Triumph Technical Services, Milton Keynes, UK). Six weeks after surgery the rats were again anaesthetised, ventilated, assessed by echocardiography, and subsequently subjected to haemodynamic measurements as previously described [20]. The rats were sacrificed 6 weeks after surgery, and only those LAD operated rats with HF (n = 20 out of 25 rats operated; Table 1) were included in the study.

**Table 1:** Animal characteristics of Sham and HF rats.

	Sham(n = 9)	HF(n = 20)
Body weight, g	380±10	374±10
Heart weight, g	1.42±0.04	2.51±0.10***
Heart weight/body weight, g/kg	3.74±0.14	6.71±0.32***
Lung weight, g	1.41±0.04	4.33±0.20***
LVEDP, mmHg	4±1.3	23±1.6***
LVSP, mmHg	101±5	88±4*
LVDd, mm	7.3±0.3	10.3±0.1
LVFS, %	54.6±1.9	13.4±0.9***
LAD, mm	4.9±0.2	8.5±0.3***

Animal characteristics are given as mean values ± SEM. LVEDP, left ventricular end diastolic pressure; LVSP, left ventricular systolic pressure; LVDd, left ventricular diameter diastole; LVFS, left ventricular fractional shorting; LAD, left atrial diameter;

* HF vs. Sham p<0.05;

*** HF vs. Sham p<0.001.

### Radioligand Binding Assay

Binding assays were performed on membranes of neonatal and adult cardiac ventricle using [^3^H]GR113808 (0.1–0.5 nM) or [^125^I]SB207710 (0.004–0.5 nM) as radioligands in the absence and presence of 5-HT (100 µM) by different methods, either essentially as described by Bach et al. [21], where bound and free radioligand were separated by filtration, or using precipitation with IgG-polyethylene glycol to separate bound from free radioligand [22]. Binding assay in isolated neonatal ventricular cardiomyocytes (1.2–1.5×10^6^ cells/well) was performed in 6-well plates. Cells were scraped in Hanks’ Balanced Salt Solution and the cell suspension filtered through Whatman GF/A filters as described previously [23].

### Real-time Quantitative Reverse Transcriptase Polymerase Chain Reaction (RT-PCR)

Foetal and neonatal cardiac ventricular tissue for RT-PCR was collected immediately after sacrifice of foetal rats 3 days (n = 6) and 1 day (n = 5) before expected birth (days -3 and -1) and neonatal rats 1 (n = 10), 3 (n = 6) and 5 (n = 6) days after birth (days 1, 3 and 5). In HF rats (n = 6) non-infarcted LV tissue (mostly septum) was collected immediately after measurement of LV weight. Corresponding LV tissue was collected from Sham (n = 5) and age matched 113-day-old normal rats (n = 5). Cardiac tissue was stored in RNAlater (Ambion). Total RNA was isolated from tissue homogenised in Trizol (1 ml/100 mg tissue, Invitrogen) with a mill homogeniser (Retch MM301, Retsch) and DNase treated (RQ1, Promega). The cDNA was synthesised from 5 µg total RNA (OD_260/280 nm_ >1.9 and RIN values >7.5) primed with oligo-dT (500 ng) using 400 U Superscript III reverse transcriptase (Invitrogen) in a 40 µl reaction at 50°C. A standard curve with 0.5–10 µg total RNA was made to control for reverse transcription and PCR quantification. Reactions without reverse transcriptase were run in parallel to control for contamination with chromosomal DNA. Sets of primers and probes were used to examine the cardiac ventricular mRNA expression of the 5-HT_4(b)_ (the most abundant cardiac 5-HT_4_ receptor splice variant [24]), 5-HT_2A_ and 5-HT_2B_ serotonin receptors, serotonin transporter (5-HTT [25]) and ANP [1], [12] in addition to both MHC-α and MHC-β mRNA [26].

The standard curve method was used to calculate individual expression level for each mRNA analysed [27]. Each target gene was normalised to the geometric mean of Arbp (acidic ribosomal phosphoprotein P0; M = 0.289), Tbp (TATA box binding protein; M = 0.355), Rpl4 (Ribosomal protein L4; M = 0.275) and Rpl32 (Ribosomal protein L32; M = 0.364) for each sample by the method of Vandesompele et al. [28] and described by Brattelid et al. [25].

The mean value of mRNA levels in 113-day-old and Sham hearts were assigned the value 1 to simplify comparison of mRNA with foetal/neonatal and HF, respectively.

### Contractility of Cardiac Muscles

Heart ventricles from foetal rats 3 days (n = 12) and 1 day (n = 9) prior to expected delivery and from rats aged 3 (n = 9), 9 (n = 12) and 20 (n = 8) days, as well as posterior left ventricle papillary muscles from HF (n = 20), Sham (n = 15) and 113–day-old (n = 13) rats were isolated and prepared for functional analyses.

Contraction-relaxation cycles (CRCs) were recorded and analysed as previously described [29] with respect to maximal developed force (F_max_) and maximal rate of force development (dF/dt)_max_. Inotropic responses to agonists were expressed as percent increases in (dF/dt)_max_. The experiments were performed in the presence of blockers (added 90 min prior to agonist) of adrenergic (α_1_, prazosin 0.1 µM, K_d_ 0.2 nM [30]; β, timolol 1 µM; K_d_ 0.8 nM [31]) and muscarinic cholinergic (atropine 1 µM, K_d_ 4.2 nM [32]) receptors. Serotonin was added to the organ bath as a single concentration (10 µM) to give a complete activation of the serotonin receptors. The response was measured after 2–7 minutes, when fully developed, as previously demonstrated [19]. To separate between different 5-HT receptor responses, the 5-HT_2A_ receptor antagonist ketanserin (0.1 µM; K_d_ 0.8 nM [33]) or the 5-HT_4_ receptor antagonist GR113808 (1 µM; K_d_ 0.09 nM [34]) or both were added 90 min prior to agonist to completely antagonise the positive inotropic response mediated through 5-HT_2A_ or 5-HT_4_, respectively [19]. Neither GR113808 nor ketanserin influenced basal contraction-relaxation cycle (CRC) characteristics or electrical stimulation threshold.

### Statistics

Data are expressed as mean ± SEM from n animals. P<0.05 was considered statistically significant. Nonparametric Mann-Whitney test was used when evaluating mRNA expression level and paired t-test when evaluating drug response.

## Results

### Characteristics of Foetal and Failing Hearts

**Figure 1:**
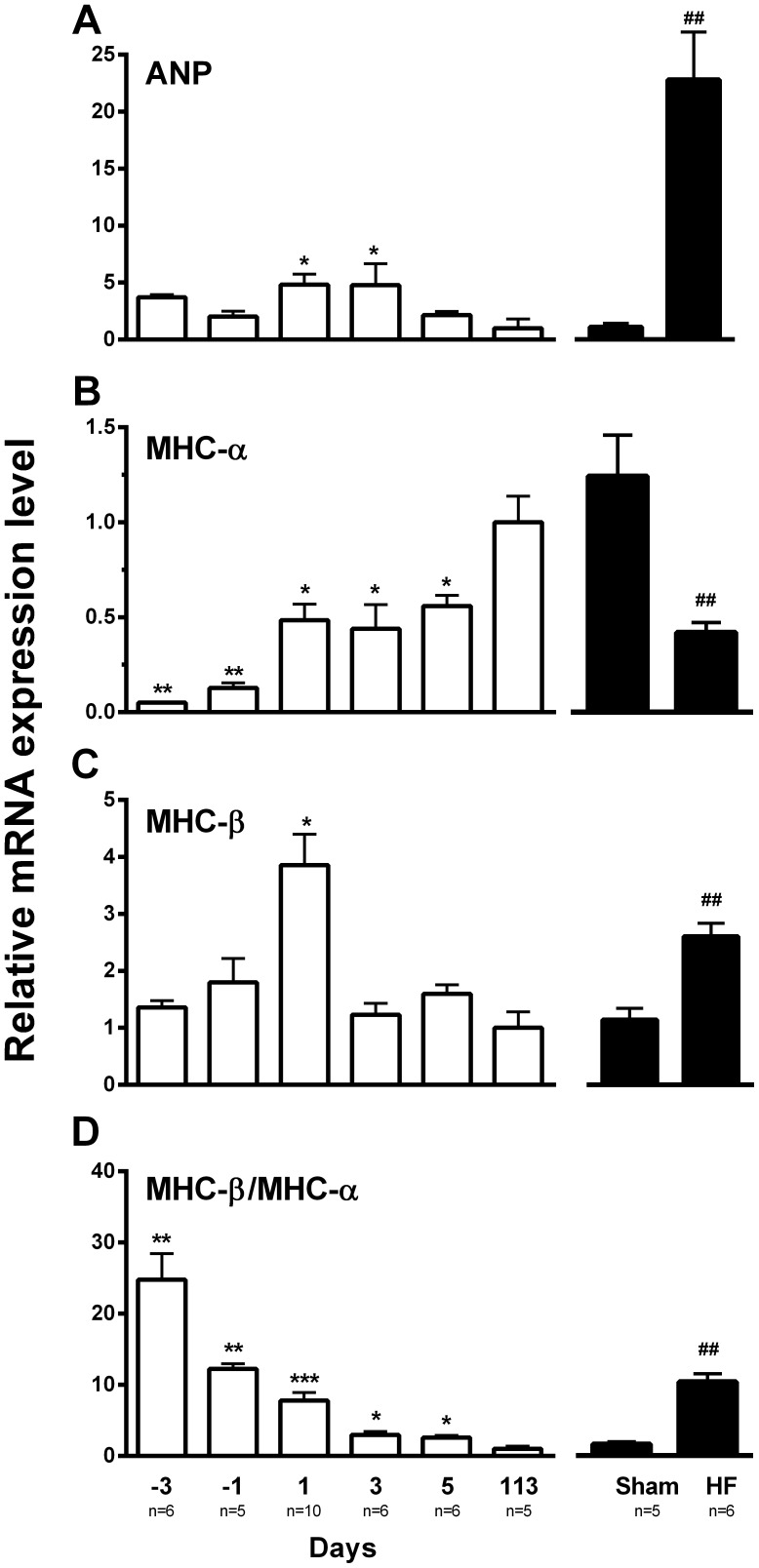
Expression profiles of mRNA markers of cardiac phenotypes. Messenger RNA expression in cardiac ventricles from foetal (day 3 and 1 before expected birth; days -3 and -1), neonatal (day 1, 3 and 5 after birth; days 1, 3 and 5), adult (day 113), Sham and HF rats. (A) Expression of ANP mRNA increases at birth and in HF; (B) MHC-α mRNA expression increases at birth and during transition from neonatal to adult and decreases in HF; (C) MHC-β mRNA expression demonstrates a transitional increase at birth and an increase in HF; (D) The ratio of MHC-β over MHC-α mRNA expression confirms transition in phenotype from foetal to adult and from Sham to HF. The results are normalised to the geometric mean of Arbp, Tbp, Rpl4 and Rpl32 and presented relative to day 113 (assigned value 1) for foetal and neonatal, and to Sham (assigned value 1) for HF. *vs. day 113 p<0.05; **vs. day 113 p<0.01; ***vs. day 113 p<0.001; ##HF *vs*. Sham p<0.01.

The expression profiles of ANP, MHC-α and MHC-β mRNA (Figure 1) are in line with previous data [12], [26], [35] indicating reactivation of a “foetal gene programme” in HF. The changes in ratio of MHC-β to MHC-α (Figure 1) are also consistent with the transition from a foetal to a neonatal phenotype as well as the development of HF [36].

Of the LAD-ligated rats, the 20 rats included in the HF group had large anterolateral infarcts and signs of congestive HF including tachypnea, pleural effusion and pulmonary congestion. Left ventricular diameter was substantially increased with reduced systolic performance (Table 1). Also, the HF rat had increased LVEDP, increased left atrial diameter and increased lung weight, all signifying congestive heart failure. For rat characteristics and haemodynamic data, see Table 1.

### Expression of 5-HT_4_ Receptor mRNA

**Figure 2:**
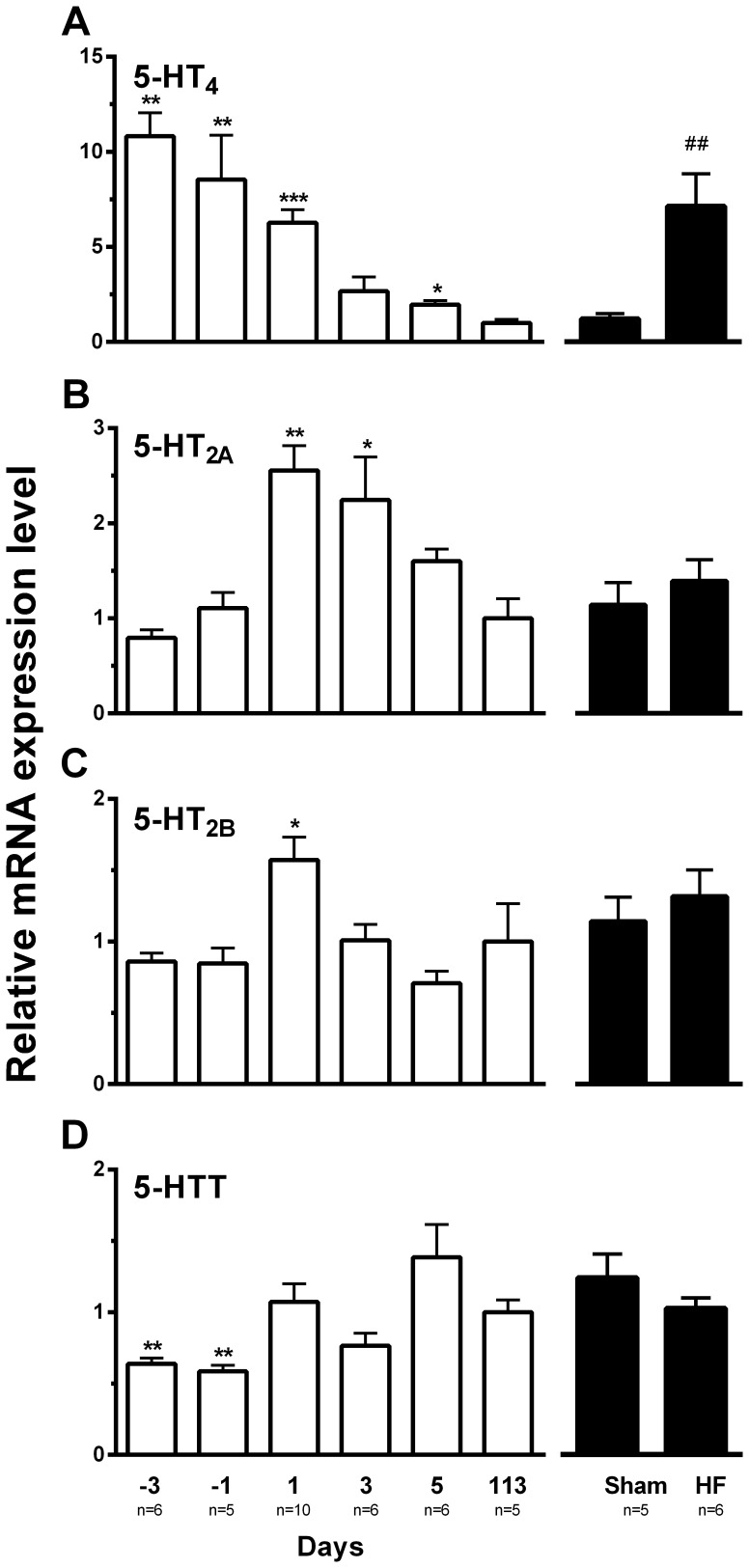
Expression of 5-HT_4(b)_, 5-HT_2A_, 5-HT_2B_ and 5-HTT mRNA in left ventricular myocardium. Messenger RNA expression in cardiac ventricles from foetal (day 3 and 1 before expected birth; days -3 and -1), neonatal (day 1, 3 and 5 after birth; days 1, 3 and 5), adult (day 113), Sham and HF rats. (A) 5-HT_4(b)_ mRNA levels decreased with foetal and neonatal development and the level in adult myocardium was one tenth of that at day -3 but reappeared in HF; (B) 5-HT_2A_ mRNA expression increased at birth (day 1), decreased during neonatal development and was unaltered in HF; (C) 5-HT_2B_ mRNA expression was transiently increased at birth (day 1) compared to adult (day 113) and not changed in HF; (D) 5-HTT mRNA expression was lower in foetal hearts compared to neonatal, adult, Sham and HF which all showed a similar expression level. The results are normalised to the geometric mean of Arbp, Tbp, Rpl4 and Rpl32 and presented relative to day 113 (assigned value 1) for foetal and neonatal or to Sham (assigned value 1) for HF. *vs. day 113 p<0.05; **vs. day 113 p<0.01; ***vs. day 113 p<0.001; ##HF *vs*. Sham p<0.01.

The 5-HT_4_ mRNA expression level decreased through foetal and neonatal development from day -3 to day 5 and reached a minimum in normal adult heart (Figure 2A). In the failing adult ventricle the 5-HT_4_ receptor expression level was increased 8-fold compared to Sham. Although the increased expression level of 5-HT_4_ in HF seems lower compared to that observed in foetal cardiac ventricle, it is rather similar to the neonatal phenotype and in line with previous observations in HF [1], [13].

**Figure 3:**
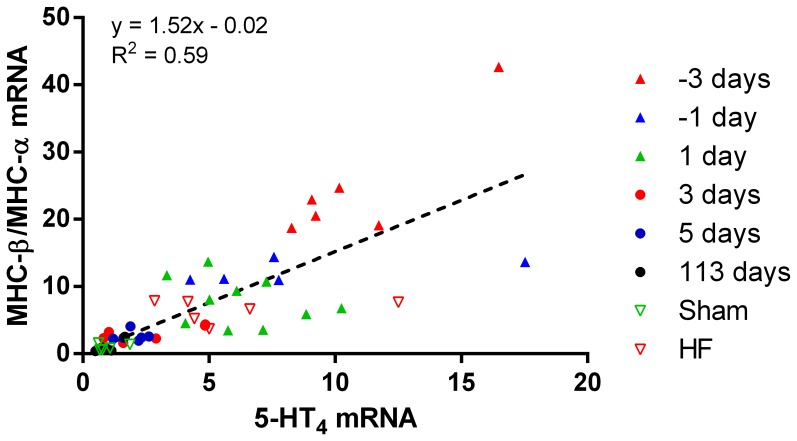
Correlation between the MHC-β/MHC-α mRNA expression ratio and the 5-HT_4_ mRNA expression level in ventricles from foetal and neonatal hearts and left ventricular papillary muscles from 113-day-old, Sham and HF rats. The ratio of MHC-β to MHC-α mRNA levels and 5-HT_4_ mRNA level showed a proportional relationship with a slope which significantly deviated from zero, p<0.0001 (linear regression analysis).

The cardiac 5-HT_4_ receptor mRNA expression was related to the MHC-β/MHC-α mRNA expression ratio, which is an indicator of cardiac development (Figure 3). Accordingly, both 5-HT_4_ mRNA and MHC-β/MHC-α mRNA expression ratio were low in cardiac tissue from postnatal, adult and Sham rats and higher in prenatal, newborn and HF rat hearts.

Binding of [^3^H]-GR113808 and [^125^I]-SB207710 to 5-HT_4_ receptors was tested with different methods and several approaches to define specific binding in neonatal ventricular preparations. In general, the total binding barely exceeded the non-specific binding, and the estimated specific binding varied between 0 and 2, or 0 and 4 fmol/mg membrane protein for [^3^H]-GR113808 and [^125^I]-SB207710 respectively, with too much variability to allow a robust quantification or further characterisation.

### 5-HT_4_-mediated Inotropic Response

**Figure 4:**
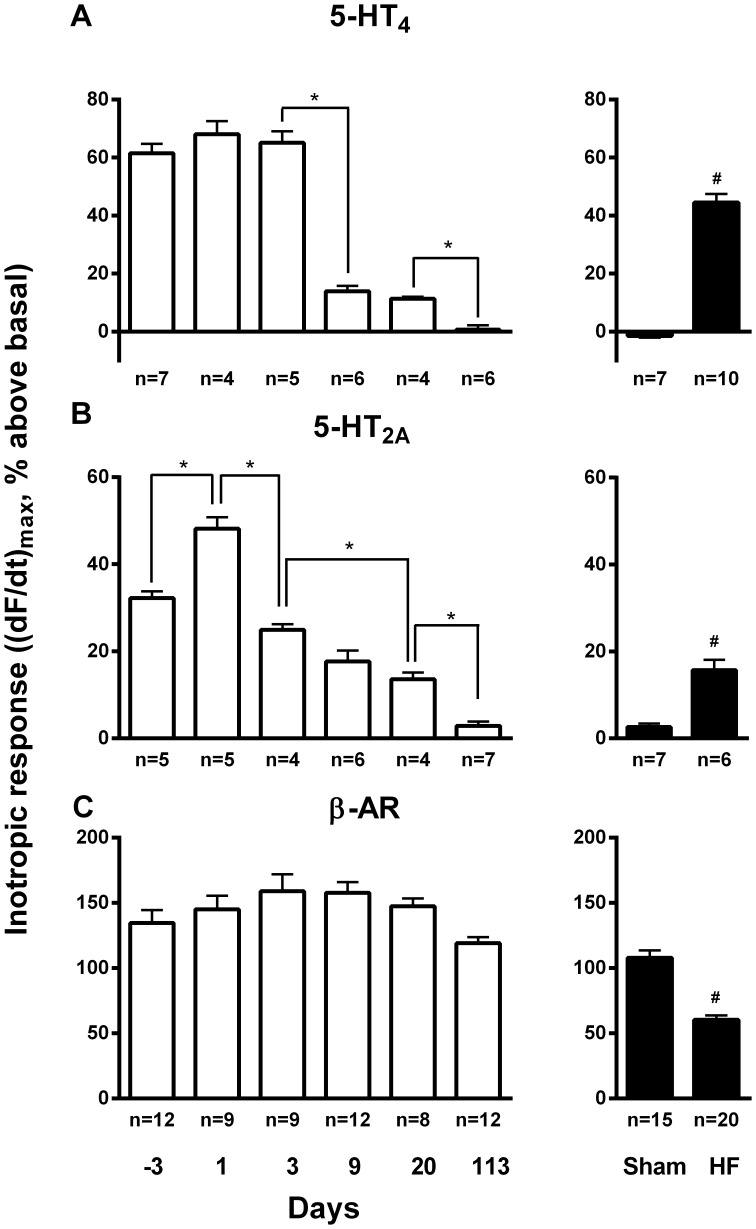
Inotropic responses to serotonin and to isoproterenol in ventricles from foetal and neonatal hearts and left ventricular papillary muscles from 113-day-old, Sham and HF rats. Positive inotropic response to serotonin (10 µM) in ventricles from foetal and neonatal rats and papillary muscles from normal adult (day 113), Sham and HF rats driven at 1 Hz in the presence of prazosine (0.1 µM), timolol (1 µM) and atropine (1 µM); ketanserin (0.1 µM; A) or GR113808 (1 µM; B) and inotropic response to subsequent addition of isoproterenol (100 µM; C). The contractile response to serotonin or isoproterenol was measured after stabilisation at its maximum within 2–7 minutes as previously demonstrated [13]. Panels show maximum inotropic response in % above basal. *vs. group indicated p<0.05; #HF *vs*. Sham p<0.05.

**Figure 5:**
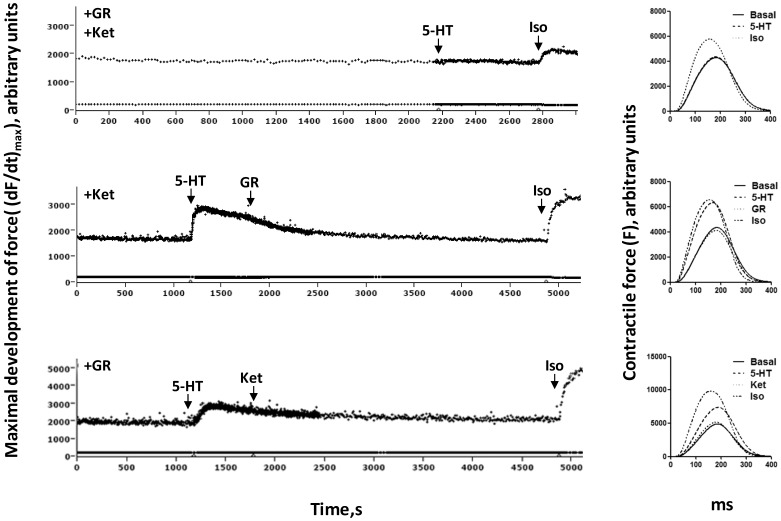
Original tracings of changes in contractility (left panels) as well as averaged contraction-relaxation cycles (right panels) illustrating the effects of 5-HT, GR113808 (GR), ketanserin (Ket) and isoproterenol (Iso) in ventricles from 3-day-old rats. *Upper left panel:* In the presence of preadded GR113808 (1 µM) and ketanserin (0.1 µM), 5-HT (10 µM) does not cause any change in contractility (measured as maximal development of force, (dF/dt)_max_). For comparison, activation of the β-adrenergic receptors (β-AR) by isoproterenol (100 µM) induces an increase in contractiity as well as a shortening of the contraction-relaxation cycle (CRC; *upper right panel*). *Middle left panel*: The 5-HT (10 µM)-induced increase in contractility in the presence of preadded ketanserin (0.1 µM) is completely antagonised by GR113808 (1 µM). *Middle right panel*: During the 5-HT-mediated increase in contractile force, the CRC is shortened as observed under isoproterenol exposure, indicating a cAMP-mediated mechanism. *Bottom left panel*: In the presence of preadded GR113808 (1 µM), 5-HT (10 µM) mediates an increase in contractility which is completely antagonised by addition of ketanserin (0.1 µM). *Bottom right panel*: The 5-HT-mediated increase in contractile force under GR113808 exposure occurs with no shortening of the CRC, indicating a non-cAMP-mediated mechanism.

Serotonin stimulation in the foetal and neonatal ventricular tissue resulted in a robust positive inotropic response (Figure 4A) which was effectively antagonised by the selective 5-HT_4_ antagonist GR113808 (1 µM, Figure 5 middle panels). However, at day 9 the 5-HT_4_-mediated inotropic response decreased dramatically and decreased further to an essentially undetectable response in left ventricle of the adult (113-day-old) animals and Sham. In CHF the 5-HT_4_-mediated inotropic response increased to levels observed immediately after birth. As previously found in HF [1], [13], the 5-HT_4_-mediated inotropic response in neonatal rat ventricle was associated with a lusitropic response (hastened relaxation), consistent with a cAMP-mediated mechanism (Figure 5 middle right panel).

### mRNA Expression and Function of the 5-HT_2A_ and 5-HT_2B_ Receptors

We observed that the ventricular 5-HT_2A_ mRNA expression transiently increased more than 2-fold at birth from the foetal level and gradually returned to this level in the adult heart (Figure 2B). In HF there was no change in the 5-HT_2A_ mRNA expression level compared to Sham. The 5-HT_2A_-mediated inotropic response, which was about half the size of the 5-HT_4_ response in foetal heart (day -3), decreased after a transient increase at birth and was almost negligible in adult non-failing and Sham ventricle (Figure 4B). In adult failing ventricle the 5-HT_2A_-mediated inotropic response reappeared to about one third of the 5-HT_4_-mediated inotropic response. As previously described in HF and hypertrophy [1], [13], the 5-HT_2A_-mediated inotropic response in neonatal rat ventricle was not associated with a lusitropic response (Figure 5 bottom right panel), consistent with a non-cAMP-dependent mechanism, probably dependent on myosin light chain phosphorylation as demonstrated in HF [13].

The ventricular 5-HT_2B_ mRNA expression increased transiently at birth but not in HF (Figure 2C). The positive inotropic response in ventricles to 10 µM 5-HT was totally abolished in the presence of concomitant 5-HT_4_ (GR113808, 1 µM; K_i_ at human 5-HT_2B_: 0.87 µM [37]) and 5-HT_2A_ (ketanserin, 0.1 µM; K_i_ at rat 5-HT_2B_: 3.56 µM [38]) blockade demonstrating the absence of any contribution from 5-HT_2B_ receptors to the inotropic response (Figure 5 upper panels).

### Development of Inotropic Effect of β-adrenergic Receptors

The inotropic response to stimulation of β-adrenergic receptors did not change significantly from late foetal through neonatal development and was also quite similar in adult heart (Figure 4C). As expected, the β-AR-mediated inotropic response decreased in the failing heart (Figure 4C) [20].

### Serotonin Transporter mRNA Levels

We observed a lower level of 5-HTT mRNA expression in the foetal stages analysed compared to adult (Figure 2D). After birth the 5-HTT mRNA expression level was rather similar in neonatal, adult and HF rat hearts (Figure 2D).

## Discussion

In this study we focused on whether the 5-HT receptor mRNA expression and function in late foetal and neonatal heart ventricle resemble those observed in HF in adult rats.

### Cardiac Regulation of 5-HT_4_ Receptor in Foetal Development and Heart Failure

The failing heart has been associated with a foetal genotype [2] and the increased 5-HT_4_ gene expression and concomitant 5-HT_4_-mediated inotropic response to serotonin previously demonstrated in the ventricles of failing rat heart [12], [13] is also observed in late foetal cardiac development (Figure 2 and Figure 3). Thus, the 5-HT_4_ mRNA expression level and the corresponding 5-HT_4_-mediated inotropic response accompany each other through the different developmental stages of the heart and in HF. The presence of robust 5-HT_4_ responses in the foetal/neonatal cardiac ventricle implies a role of the 5-HT_4_ receptor in cardiac development. The importance of their re-expression in failing ventricle has been evaluated in other studies [13], [26].

Other lines of evidence also suggest a functional role of the 5-HT_4_ receptor in the foetal heart. Offspring of female mice immunised with a peptide corresponding to the second extracellular loop of the human 5-HT_4_ receptor developed foetal and neonatal arrhythmia, as well as ataxia [39]. However, the impact of this 5-HT_4_ receptor autoimmunity on cardiac function remains uncertain.

Neonatal cardiomyocytes are an attractive model system to characterise several aspects of cardiac function at the cellular level [40], [41], [42]. Importantly, since neonatal rat hearts express functional 5-HT_4_ receptors linked to inotropic effects, it should be further evaluated whether neonatal rat cardiomyocytes may serve as a useful model for cardiomyocytes from failing rat hearts as well as from the less available failing human hearts in studies of cardiac 5-HT_4_ receptor function.

### Cardiac Regulation of 5-HT_2A_ and 5-HT_2B_ Receptors in Foetal Development and Heart Failure

Pathways activated by G_q_/G_11_-coupled receptors play essential roles in the development of cardiac hypertrophy [43]. The G_q_-coupled 5-HT_2A_ and 5-HT_2B_ [44] found in cardiomyocytes [44], [45], [46] and non-cardiomyocytes [12], [47] could mediate cardiac growth and hypertrophy in cardiac development as well as in heart failure.

In the hypertrophic heart we have reported an increase in 5-HT_2A_ mRNA expression and 5-HT_2A_-mediated inotropic response [12]. 5-HT_2A_ might therefore be part of a cardiotrophic mechanism. The transient increase in ventricular 5-HT_2A_ mRNA and 5-HT_2A_ receptor-mediated inotropic responses around birth resembles previous observations in acute HF [19], a pathological condition with an acute increase in wall stress. At the time of birth the heart is also exposed to an acute increase in wall stress associated with the circulatory changes around birth [48]. It is therefore tempting to suggest that the 5-HT_2A_ receptor might play an important role in response to the acute increase in wall stress observed at birth and in acute heart failure. In chronic HF however, there is an increase in the 5-HT_2A_-mediated inotropic response (Figure 4B) which does not coincide with an increase in the 5-HT_2A_ mRNA expression (Figure 2B). Thus chronic HF might induce other regulatory mechanisms which increase 5-HT_2A_-mediated contractility without increasing the mRNA level.

The coincidence of the increase in 5-HT_2B_ mRNA at time of birth associated with an increased left ventricular output [48] can suggest a possible involvement of non-cardiomyocyte localised 5-HT_2B_ receptors in development and progression of cardiac hypertrophy as suggested previously [12]. Jaffrè et al [49] revealed that the 5-HT_2B_ receptor in cardiac non-cardiomyocytes is pivotal in angiotensin and adrenergic receptor mediated hypertrophy. This was supported by recent findings where the 5-HT_2B_ antagonist SB204741 had anti-hypertrophic effects in an iso-proterenol-induced rat hypertrophy model [50].

Even though GR113808 and ketanserin have some affinity at 5-HT_2B_ receptors, at the concentrations used in the contractility assay they would not block these receptors significantly in the presence of 10 µM 5-HT, which is about 1000-fold the EC_50_ value of 5-HT at human 5-HT_2B_ receptors (8.95 nM according to Wainscott et al [51]). Thus, a 5-HT_2B_-mediated inotropic response to 5-HT would have been demonstrated, if present.

### Cardiac Regulation of β-adrenergic Receptor in Foetal Development and Heart Failure

Although the β-adrenergic receptor density has been shown to be regulated during foetal and neonatal development [52] the functional β-adrenergic response did not show significant changes during late foetal and postnatal development (Figure 4C).

### Cardiac Regulation of Serotonin Transporter in Foetal Development and Heart Failure

The role of 5-HT receptors in cardiac tissue will depend on the availability of endogenous 5-HT, most likely determined by the plasma level of 5-HT and 5-HTTs present locally. The trophic 5-HT signal in foetal cardiomyocytes has been shown to be concentration dependent (with an optimum at 4 µM) under control of the 5-HTT activity [7]. There is evidence for an increase in plasma level of 5-HT in coronary artery disease and events [14] and in hypertension [53]. Inhibiting the 5-HTT with a serotonin reuptake inhibitor in Sham and failing hearts did not alter the inotropic response (data not shown). A functional role of 5-HTT in the failing heart thus remains elusive.

### Limitations

The data presented here is based on functional observations of the inotropic response in cardiac tissue due to pharmacological stimulation and inhibition of the 5-HT_4_ receptor. Demonstration of the 5-HT_4_ receptor protein expression in the cardiac tissue would also be of interest. With both [^3^H]GR113808 and [^125^I]SB207710 we were not able to detect specific binding corresponding to 5-HT_4_ receptors in the cardiac ventricle tissue samples analysed. This is in line with our inability in previous studies to quantify specific 5-HT_4_ receptor levels in failing cardiac ventricle [12], [13]. In the human heart the expression of the 5-HT_4_ receptor is very low (<4 fmol per mg protein in right atrium) [54]. An even lower level of the 5-HT_4_ receptors in the ventricle and possible species differences complicates the ability to demonstrate the presence of cardiac 5-HT_4_ receptors with currently available methods. Although the ability to label new 5-HT_4_ receptor antagonists with even higher selectivity and affinity for the 5-HT_4_ receptor [55] and lower non-specific binding might improve future binding assays, the density of 5-HT_4_ receptors in cardiac ventricle will remain low and likely near the limit of proper quantification. In a previous publication [56] we have demonstrated similar slopes of standard curves used to determine 5-HT_4_ mRNA expression in hippocampus and cardiac left ventricle. The difference in crossing point were ∼8 Cp’s, representing approximately 250-fold difference in mRNA level between the hippocampus and heart [56]. Hippocampus expresses about 120 fmol 5-HT_4_ receptors per mg protein [57]. Assuming similar ratios between mRNA and protein, the corresponding protein level of the 5-HT_4_ receptor in the heart can be estimated to about 0.5 fmol per mg protein, which would be below the limit of detection with the radiolabeled ligands available. Since 5-HT_4_ receptor protein is clearly below the level of detection with available radioligand binding methods, the presence of the protein and its level of expression in this and previous studies had to be inferred from its functional effects on contractility. Since 5-HT_4_ receptor stimulation in previous studies (e.g. [13], [58]) was shown to produce a submaximal inotropic response compared to beta-adrenoceptor stimulation, a reasonable correlation between receptor expression levels and the maximal 5-HT_4_-mediated inotropic response can be inferred, and we are therefore interpreting an increasing maximal 5-HT_4_-mediated inotropic response to reflect increasing 5-HT_4_ receptor protein levels.

At present nine 5-HT_4_ receptor splice variants differing in the C-terminus have been described [59]. We have only monitored the gene expression of the most abundant 5-HT_4_ receptor splice variant 5-HT_4(b)_ [24]. It might therefore be possible that other 5-HT_4_ receptor splice variants are regulated and contribute to the total 5-HT_4_ receptor level and effects during cardiac development and CHF.

### Conclusions

Our findings that the 5-HT_4_ receptor mRNA is expressed at high levels in foetal and failing hearts with corresponding changes in the inotropic responses suggest a role of the 5-HT_4_ receptor in heart development and failure. We also demonstrated that the 5-HT_2A_ receptor mRNA and function are transiently increased at birth, whereas the 5-HT_2B_ receptor mRNA expression is also transiently expressed at birth, but without any effect on cardiac contractility.
